# Investigating the relation between social media, dating app use and body image dimensions: A cross‐country study

**DOI:** 10.1111/bjhp.70067

**Published:** 2026-03-30

**Authors:** Gianluca Lo Coco, Rachel Rodgers, Emily A. Harris, Charlotte Markey, Alvaro Sicilia, Annie Aimé, Jacinthe Dion, Laura Salerno, Naomi Hayami‐Chisuwa, Hannah J. White, Carolyn R. Plateau, Antonio Granero‐Gallegos, Christophe Maïano, Gian Mauro Manzoni, Giada Pietrabissa, Catherine Bégin, Marie‐Éve Blackburn, Esben Strodl, Matthew Fuller‐Tyszkiewicz, Marita McCabe

**Affiliations:** ^1^ University of Palermo Palermo Italy; ^2^ North Eastern University Boston Massachusetts USA; ^3^ University of Melbourne Melbourne Victoria Australia; ^4^ Rutgers University Camden New Jersey USA; ^5^ University of Almeria Almeria Spain; ^6^ Université du Québec en Outaouais (UQO) Saint‐Jérôme Quebec Canada; ^7^ Université du Québec à Trois‐Rivières Trois‐Rivières Quebec Canada; ^8^ Osaka City University Osaka Japan; ^9^ Loughborough University Loughborough UK; ^10^ University of Pavia Pavia Italy; ^11^ Catholic University of Milan Milan Italy; ^12^ Laval University Quebec Quebec Canada; ^13^ ECOBES‐Research and Transfer Jonquiére Quebec Canada; ^14^ Queensland University of Technology Kelvin Grove Queensland Australia; ^15^ Deakin University Burwood New South Wales Australia; ^16^ Swinburne University Hawthorn Victoria Australia

**Keywords:** body appreciation, body image, dating apps, drive for muscularity, romantic relationships, social media, thin ideal

## Abstract

**Objectives:**

Social media emphasize appearance ideals, and their use may be associated with body dissatisfaction. To date, however, data on the relationships specific to certain platforms such as dating apps are scarce, as are cross‐country comparisons and studies including men. Thus, this study examined the relationship between social media and dating app use and body image among young adults across eight countries and considered gender and country as potential moderators.

**Methods:**

A sample of 5933 young adults (mean age = 21.54 years; 67.6% female) from Australia, Belgium, Canada, China, Italy, Japan, Spain and the United States completed an online survey. Mixed‐effects regression models tested the effects of time on social media and dating apps on body image outcomes.

**Results:**

Longer time on social media was associated with lower body satisfaction and body appreciation, and a higher drive for leanness, thin‐ideal internalization and appearance comparison. Longer time spent on dating apps was associated with lower body appreciation and appearance comparison, and a higher drive for muscularity. The associations between time spent on social media, dating apps and drive for muscularity were stronger for men than for women. Few variations across countries emerged.

**Conclusions:**

Greater social media and dating app use may be related to poor body image and related indicators across countries, particularly among young men.

**Statement of Contribution**

**What is already known on this subject?**

- The relationship between higher use of social media and poorer body image is well established.
- Some support exists for the association between body image dimensions and dating apps use.

**What does this study add?**

- More time spent on social media and dating apps may be linked to greater body image indicators in eight countries.
- The associations between time spent on social media, dating apps and drive for muscularity are stronger for men than for women.

## INTRODUCTION

Social media has increasingly become an important aspect of daily life for many, and its use has consistently increased among emerging adults within the last decade (Pew Research Center, 2024). Eighty‐four per cent of young adults in the United States report using social media sites, such as Facebook, Instagram, Snapchat and TikTok (Pew Research Center, 2021). As highly visual forms of media, many social media platforms are relevant to developing body image and communicating strong messages regarding the centrality of appearance (Sharma & Vidal, 2023). Research has extensively supported the relationships between greater social media use and higher body image concerns, particularly in the context of highly visual platforms that convey idealized content (Holland & Tiggemann, 2016; Lo Coco & Rodgers, 2025; Markey et al., 2024; Rodgers & Rousseau, 2022). Although accumulating research has documented the relationship between the use of highly visual social media and negative body image indicators (Sharma & Vidal, 2023), this literature is limited by its primary focus on these relationships among women in English‐speaking countries (Fardouly & Vartanian, 2016; Vlasak et al., 2025). Further, the majority of the extant research has focused on social media use across platforms or within types of platforms (such as photo vs. text based) (Lo Coco & Rodgers, 2025); yet some research suggests that certain types of platforms in particular, such as dating apps, may be particularly strongly linked with body image dissatisfaction and disordered eating (Portingale et al., 2022). The aim of this study was therefore to explore the relationships among social media and dating app use on the one hand, and body image dimensions on the other hand, among female and male young adults from eight different countries.

The media have been identified as one of the loudest purveyors of unrealistic appearance ideals (Levine & Murnen, 2009). Socially constructed appearance ideals are, by design, unrealistic and unattainable for most individuals without extensive investment in products and services, often at the expense of physical and mental health (Rodgers et al., 2023). These ideals are gendered, with an emphasis on extreme thinness and a small, toned body for women and lean hyper‐muscularity among men. Given that one of the main features of social media is the capacity to share photos and videos, most users are exposed to unrealistically attractive images of peers, influencers and celebrities, which may induce social comparisons that result in feelings of body dissatisfaction (Fardouly et al., 2017; Verduyn et al., 2020). For example, it has been suggested that exposure to Instagram images that show attractive and thin celebrities and peers are associated with higher body dissatisfaction levels (Brown & Tiggemann, 2016; Tiggemann et al., 2018). However, body image is a multidimensional construct referring to the thoughts, feelings and perceptions related to one's body and embodiment (i.e., the experience of inhabiting one's body) (Thompson & van den Berg, 2002), and there is evidence that the association between media use and body image varies across certain dimensions (Grabe et al., 2008; Mingoia et al., 2017). Recent meta‐analytic findings (Saiphoo & Vahedi, 2019) evidenced a small relationship between greater social media use and higher body image disturbance, with the strongest effect sizes for the cognitive (e.g., internalization of the thin‐ideal, that is endorsement of appearance ideals as a personal standard) and behavioural (e.g., disordered eating) dimensions of body image. Another meta‐analysis of experimental studies reported that exposure to thin‐ideal images had a moderately detrimental impact on body image compared to appearance‐neutral conditions (de Valle et al., 2021). This review adopted a broad definition of body image, encompassing dimensions such as body dissatisfaction, behavioural symptoms, body esteem and drive for thinness. However, the effects were small when outliers were removed, and the effect sizes of the longitudinal correlations with body image were very small. Thus, the relationship between higher use of social media and poorer body image is now well established but with a small effect size.

Gender has been examined as a relevant moderator of the relation between social media use and body image concerns, since appearance pressures tend to be stronger for women than men (Gillen & Markey, 2023). The meta‐analysis by Saiphoo and Vahedi (2019) on the association between social media use and body image disturbance showed that studies with higher proportions of women did not have larger effect sizes, suggesting that this relationship may not be different across men and women. The meta‐analysis conducted by de Valle et al. (2021) revealed that the proportion of female subjects in the sample did not moderate the negative impact of exposure to appearance‐ideal images on body image in experimental studies, nor did it moderate the effect of exposure to appearance‐ideal images in higher risk social media contexts on body image. The most recent review on social media use and body dissatisfaction (Vlasak et al., 2025) also looked at gender of samples (mixed vs. male vs. female) and found no significant differences. Nevertheless, the lack of documented gender differences may be attributable to the underrepresentation of male participants in research concerned with body image, with a consequent overreliance on data from female participants, in conjunction with the predominant use of constructs and measures related to body image that are gendered and tend, for example, to focus on thinness as opposed to muscularity. Furthermore, it may also result from characteristics of the country in which the data were collected. The large majority of studies have investigated relationships between social media and body image in one specific country, and cross‐national comparisons are widely missing. It is likely that differences in cultural values, beauty standards and media landscapes across countries can influence how social media use affects body image. It was shown that appearance ideals that are gendered, heteronormative, ageist and Eurocentric contribute to centring concerns around weight, muscularity, attractiveness and fairness (Rodgers et al., 2023). Cross‐cultural studies have demonstrated that in a multitude of non‐Western countries, the adoption of a Western lifestyle and the social shift from collectivistic to individualistic values have the potential to engender an increase in body image concerns (Swami et al., 2010; Wardle et al., 2006), continuing to support the importance of sociocultural models of body image.

Recent studies have indicated that dating app use may be related to outcomes pertaining to appearance, in a manner analogous to that of other social media platforms (Bowman et al., 2025). Dating apps have become an increasingly popular type of digital platform, with some 48% of young people aged 18–29 years from the US reporting having ever used a dating app (Pew Research Center, 2023) such as Tinder, Grindr or Bumble. Location‐based smartphone dating apps have been increasing in popularity given the possibilities of tapping into a pool of potential partners (Sumter & Vandenbosch, 2019), and they allow users to access online dating anywhere and anytime (Jung et al., 2019; Sumter et al., 2017). Prior research suggests that men appear to be the most active on dating platforms, as compared to other genders (Abramova et al., 2016; Blanc, 2023; Castro & Barrada, 2020; Sumter & Vandenbosch, 2019).

Although dating apps place a central role on appearance, quantitative research examining the link between body image and online dating is limited relative to the larger literature on social media (Konings et al., 2022). Dating apps offer a great deal of flexibility in how people present themselves to potential partners, for example, by exaggerating aspects of their appearance to make themselves more attractive (Ellison et al., 2012; Whitty, 2008). Despite the potential for both social media apps broadly and dating apps in particular to exert a negative influence on body image, there are also some distinctive characteristics and particularities of dating apps. For example, dating apps introduce an additional dimension of direct evaluation and competition, while most social media platforms primarily focus on the presentation of an idealized self and the comparison to broader social trends. Thus, while on general social media users may be exposed to unrealistic beauty standards (Bonfanti et al., 2025; Fioravanti et al., 2022; Holland & Tiggemann, 2016), those utilizing dating applications specifically may encounter an additional pressure to present their own bodies in a particular, frequently edited manner, to better align with body ideals (Tran et al., 2020; Waling et al., 2023). This may precipitate heightened self‐scrutiny, rejection sensitivity and body shame (Blake et al., 2022; Rodgers, Campagna, et al., 2020). Thus, dating app users may be susceptible to self‐objectification, as evidenced by a propensity to place increased emphasis on profile pictures and body appearance (Strubel & Petrie, 2017). In turn, this may subsequently precipitate body dissatisfaction and concerns regarding one's physical appearance (Bell et al., 2018; Bowman et al., 2025). It is also noteworthy that heterosexual women and men typically engage with the opposite sex on dating apps without engaging in a comparison of their bodies with those of the opposite sex. In contrast, the phenomenon of appearance comparison on social media has been identified as a catalyst for the emergence of body image concerns (Bonfanti et al., 2025; Holland & Tiggemann, 2016).

To date, a growing number of studies have provided empirical support for the association between body image indicators and dating apps use. A recent review showed that 19 studies reported a significant negative association between dating app use, primarily in terms of frequency and duration of usage, and body image variables (Bowman et al., 2025), such as body dissatisfaction, muscularity dissatisfaction, body fat dissatisfaction, body shame, body esteem and body surveillance. However, some studies have failed to replicate the relationship between dating app use and poorer body image. For example, a study that used Ecological Momentary Assessment methodology to assess eating disorder symptoms throughout the day (Portingale et al., 2022) revealed that women who had used dating apps at least once during their lifetime reported greater daily urges for binge‐eating/purging. However, they did not report increased negative feelings towards their body shape or weight. Similarly, in a recent study among young men with loss of control eating episodes, neither dating app nor social media use was associated with body dissatisfaction (Kelly et al., 2023). It is worth noting that the findings on gender differences in the use of dating apps are similarly inconclusive to those pertaining to social media usage. For example, associations between higher levels of dating app use and greater thin‐ideal internalization, appearance comparisons, body shame and body dissatisfaction have been reported among Tinder users, regardless of gender (Strubel & Petrie, 2017). Moreover, frequent dating app use was found to be related to body shame among men but not women, whereas dating app use was associated with body dissatisfaction among women who reported negative affect after using dating apps (Rodgers, Campagna, et al., 2020). However, this past research is limited in important ways. The majority of work in this area has focused on girls and women (Bowman et al., 2025). Additionally, only a paucity of studies has evaluated the link between body image variables and both social media and dating app use among the same participants in order to examine if the gender distribution plays a different role in different online platforms. Finally, it is worth noting that both gender and cross‐country differences in the association between social media and dating app use and body image variables have also generally not been investigated, despite sociocultural influences having been shown to be related to appearance concerns, most particularly, pressure from peers, dating partners and family (McCabe et al., 2019). The meta‐analysis by Saiphoo and Vahedi (2019) on the relation between social media use and body image showed that studies conducted in Australia had higher effect sizes than those conducted in Europe, North America and Asia. More recently, the meta‐analysis by Vlasak et al. (2025) on the association between social media use and body dissatisfaction found no significant differences between countries (European vs. Asian vs. Australian/Oceanic), but with a small number of studies included in the analysis. The most recent systematic review on the relationship between dating app use and body image did not explore cross‐country differences. This was due to the fact that the majority of the studies included in the review were conducted in Western regions and focused on white samples (Bowman et al., 2025). In summary, cross‐country comparisons on the relation between body image and the use of both social media and dating apps are lacking, and this has resulted in ignoring the contextual factors on the regional level.

### The current study

The present study aimed to enhance our comprehension of the potential interrelations between the utilization of social media and dating applications, on the one hand, and body image, on the other. To this end, it addressed the limitations of extant literature in a number of different ways. Firstly, it reported on body image in a large cross‐country sample of young adults (18–30 years), an age group susceptible to appearance concerns and vulnerable to disordered eating (Arnett et al., 2014). Given the research above, it was hypothesized that (Hypothesis 1) young adults who spend more time on social media would report poorer body image (i.e., lower body satisfaction and body appreciation, and higher internalization of the thin ideal, higher drive for muscularity and leanness, higher appearance comparison) compared to those who spend less time on social media. It was also hypothesized (Hypothesis 2) that young adults who spend more time using dating apps would report poorer levels of body image compared to those who spend less time on dating apps. Secondly, given the inconsistencies of previous research findings on gender and country differences on these relationships, the present study reported data from women and men from eight different countries to examine whether gender and regions moderate our hypothesized effects. Finally, as noted above, variations have been identified in the magnitude of the relationships between social media and dating app indices and body image across dimensions of body image (Bowman et al., 2025; de Valle et al., 2021; Saiphoo & Vahedi, 2019). In line with previous work, the present study considered a broad spectrum of dimensions of body image, with an additional focus on capturing dimensions relevant to men and that are grounded in weight‐neutral models, including body satisfaction, body appreciation, drive for leanness, drive for muscularity, appearance comparison and thin‐ideal internalization. The choice of measures was designed to include indices of positive body image that have received scant attention in the extant literature focused on the relationships between social media use and body image as well as dimensions relevant across gender, with both their cognitive and behavioural dimensions.

## METHODS

### Participants and procedures

A community sample of young adults (age range 18–30 years) was recruited via social media, online undergraduate forums and across the university settings of the recruitment sites (see McCabe et al., 2019, for a full description of the recruitment procedure). In order to enhance equitable participation across gender, the recruitment materials emphasized the significance of participation in the survey for individuals of all gender identities. The data collection process was conducted over the period from July 2018 to March 2019. A power analysis estimated that a target sample of 600 participants per country will ensure sufficient sample size from both global and local model fit perspectives within‐country to run the analyses designed to test the proposed model (McCabe et al., 2019). A total sample of 6272 participants (mean age = 21.54, SD = 3.13) was retained across eight countries: Australia (*n* = 597), Belgium (*n* = 618), Canada (*n* = 768), China (*n* = 669), Italy (*n* = 661), Japan (*n* = 622), Spain (*n* = 821) and the United States (*n* = 1516). In the current study, 5933 respondents answered questions about their use of social media and dating apps (See Table 1). The majority of participants identified as female (*N* = 4003, ranging from 58.5% to 84.9% across countries) and university students (ranging from 73.1% to 99% across countries). Given the small subsample of non‐binary participants (*N* = 92), we did not include them in the study analyses. Appropriate ethical and institutional approvals were obtained at all recruitment sites, and all participants provided informed consent.

**TABLE 1:** Social media and dating app use and time by country (*N* = 5933).

Country	*n*	Use (% yes)	Time spent M (SD)
Social media use			
Australia	597	99	3.72 (1.33)
Belgium	618	99	3.46 (1.22)
Canada	768	99	3.37 (1.17)
China	655	63	2.36 (1.18)
Italy	657	96	3.60 (1.31)
Japan	302	96	2.98 (1.49)
Spain	820	98	3.65 (1.39)
USA	1516	97	3.32 (1.27)
Dating app use			
Australia	597	21	1.68 (.98)
Belgium	618	15	1.41 (.71)
Canada	768	18	1.46 (.72)
China	655	45	2.02 (.86)
Italy	657	5	2.79 (1.60)
Japan	302	12	1.77 (1.00)
Spain	820	5	1.71 (1.06)
USA	1516	25	1.54 (.85)

*Note*: Mean value on the scale: 1 = <60 min, 2 = 1–3 h, 3 = 4–6 h, 4 = 7–13 h, 5 = 14–20 h and 6 = >21 h.

### Measures

Two single dichotomous items were used to assess both social media and dating apps use. Participants were asked: ‘Do you have accounts with any social media platforms? (e.g., Facebook, Instagram, Twitter, Snapchat, Tumblr, Periscope)’, and ‘Do you have accounts with any online platforms for dating? (e.g., Tinder, Bumble, Grindr)’. Items are scored as yes = 1 or no = 0. Regarding time spent on social media and dating apps, participants were asked: ‘If yes, how much time do you spend on social media in total per week?’, and ‘If yes, how much time do you spend using online dating platforms in total per week?’ Items are scored on a 6‐point Likert scale with the following response options: 1 = <60 min, 2 = 1–3 h, 3 = 4–6 h, 4 = 7–13 h, 5 = 14–20 h and 6 = >21 h. These brief and simple measures were adopted in line with the findings of previous studies, which operationalized social media exposure in terms of intensity of use, as weekly or daily amount of use (Fox & Vendemia, 2016; Griffiths et al., 2018; Mabe et al., 2014; Mahalingham et al., 2023; Riehm et al., 2019; Sidani et al., 2016).

The Body Appreciation Scale‐2 (BAS‐2; Tylka & Wood‐Barcalow, 2015) is a 10‐item tool that measures body appreciation (e.g., ‘I respect my body’; ‘I am comfortable with my body’), with a 5‐point response scale ranging from 1 (never) to 5 (always). The BAS‐2 items were summed to compute a total score. Measurement invariance for the BAS‐2 across countries has been previously supported (Aimé et al., 2020; Swami et al., 2023). Moreover, its one‐factor structure was supported with young adult samples (Aimé et al., 2020). Differences across gender identities were reported as negligible‐to‐small (Swami et al., 2023; Tiggemann, 2015). In the current study, the BAS‐2 showed excellent internal consistency (Cronbach's α ranges from .92 to .96 across countries).

The Body Areas Satisfaction Scale (BASS) of the Multidimensional Body‐Self Relations Questionnaire (MBSRQ) (Cash, 1990) was used to measure body satisfaction. Its nine items assess the degree of satisfaction/dissatisfaction with different aspects of one's appearance (i.e., face, hair, lower torso, mid torso, upper torso, muscle tone, weight and height) as well as overall appearance, on a 5‐point Likert‐type scale, ranging from 1 (very dissatisfied) to 5 (very satisfied). In this study, the nine BASS items were averaged to compute a mean score. Its measurement invariance across countries has been previously supported (Aimé et al., 2020) with significant improvement of model fit obtained by correlating residual variances. With regard to gender, young adult women tend to score lower on body satisfaction than men (Aimé et al., 2020). In the current study, the BASS showed a good internal consistency (Cronbach's α ranges from .77 to .88 across countries).

The Physical Appearance Comparison Scale (PACS; Thompson et al., 1991) is a 5‐item questionnaire measuring appearance comparison frequency (e.g., ‘At parties or other social events, I compare my physical appearance to the physical appearance of others’ and ‘In social situations, I sometimes compare my figure to the figures of other people’), on a 4‐point scale, ranging from 1 (never) to 4 (always). Scores for this measure are obtained by summing the participant's responses. Scores have been shown to be reliable and valid in college samples (Thompson et al., 1991). Moreover, its unidimensional factor structure and its measurement invariance across countries has been previously supported (Aimé et al., 2020). With regard to gender, previous cross‐country research suggested that women report more physical appearance comparison than men (Aimé et al., 2020). In the current study, the PACS showed acceptable internal consistency (Cronbach's α ranges from .66 to .83 across countries).

The Drive for Leanness Scale (DLS; Smolak & Murnen, 2008) is a 6‐item measure assessing the pursuit of a lean body (e.g., ‘My goal is to have well‐toned muscles’), on a 6‐point Likert scale that ranges from 1 (never) to 6 (always). Its unidimensional factor structure provided a satisfactory model fit in young adult samples and its measurement invariance across countries has been supported (Sicilia et al., 2020). With regard to gender, women tended to report lower scores in DLS than men (Sicilia et al., 2020). In the current study, the DLS showed a good internal consistency (Cronbach's α ranges from .84 to .89 across countries).

The Drive for Muscularity Scale (DMS; McCreary et al., 2004) is a 15‐item measure assessing attitudes and behaviours that reflect individuals' excessive concern with muscularity (e.g., ‘I think I would feel stronger if I gained a little more muscle mass’), on a 6‐point rating scale that ranges from 1 (never) to 6 (always). Its measurement invariance across countries has been previously supported in young adults (Sicilia et al., 2020) but with a modified model showing structural equivalence across countries. Previous studies showed that women tend to report lower scores in DMS (Edwards et al., 2014; Sicilia et al., 2020). In the current sample, the DMS showed a good internal consistency (Cronbach's α ranges from .83 to .94 across countries).

The 5‐item subscale (i.e., the Internalization Thin/Low Body Fat) from the Sociocultural Attitudes Towards Appearance Questionnaire‐4 (Schaefer et al., 2015) was used to assess the internalization of the thin ideals (e.g., ‘I want my body to look very thin’). Items are scored on a 5‐point Likert scale ranging from 1 (definitely disagree) to 5 (definitely agree). This scale good showed a good convergent validity with measures of body image and eating disturbance among adults in Western primarily college samples (Schaefer et al., 2015). Its measurement invariance across countries has been previously supported (Rodgers, Fuller‐Tyszkiewicz, et al., 2020). Previous research showed that women tend to present higher levels of thin/low body fat internalization than men across countries (Rodgers, Fuller‐Tyszkiewicz, et al., 2020). In the current sample, the SATAQ Internalization Thin/Low Body Fat scale showed good internal consistency (Cronbach's α ranges from .81 to .88 across countries).

Body mass index was used as a measure of weight status. Participants reported their weight and height and indicated the metric they were using (e.g., inches or centimetres).

### Data analyses

Our preliminary analyses were largely descriptive and estimated how many participants use social media and dating apps and how often. We calculated the proportion of young adults who use social media and dating apps (yes/no) and the time spent using social media and dating apps by gender and country.

To test Hypothesis 1—more time on social media will be associated with poorer levels of body image—we ran a series of mixed‐effects regression models (*N* = 5895). Given that most available research on the association between social media use and body image controlled for BMI (Rounsefell et al., 2020), the models controlled for BMI and age and included a random intercept for country. Continuous predictor variables were group‐mean centred. The primary predictor variable of interest was time spent on social media. Study outcomes were body satisfaction, body appreciation, drive for muscularity, drive for leanness, internalization of the thin ideal and appearance comparisons. We ran three models for each of our dependent variables. Model 1 assessed the main effect of time spent on social media on the dependent variables. Model 2 included an interaction term between time spent on social media and gender (the variable ‘male’ was coded as 1 and ‘female’ as 2). Model 3 included an interaction term between time spent on social media and country. Following up on a significant effect of country could require 28 possible pairwise comparisons. We reduced the number of comparisons by using the homogenous subsets approach. First, we ran pairwise comparisons to identify homogenous subsets of countries (groups of countries which did not significantly differ from each other on the outcome variable). As a hypothetical example, if three countries scored high on body satisfaction and did not significantly differ from each other, they were grouped as ‘High’ in body satisfaction. If the remaining five countries scored significantly lower on body satisfaction than the countries in the ‘High’ group, but did not significantly differ from each other, they were grouped as ‘Low’. Second, we tested for an interaction between social media use and the reduced country grouping variable. We used the homogenous subsets approach to aid in interpretation and minimize the number of comparisons in the final analysis. This approach also reduced the likelihood of Type I error by limiting the number of post‐hoc pairwise comparisons across countries. While our large sample size increased statistical power (i.e., reduced the likelihood of Type II error), it also meant that even small effects could reach statistical significance (Benjamin et al., 2018). Accordingly, the use of homogenous subsets helped minimize the number of comparisons and reduced the potential for spurious findings due to multiple testing.

To test Hypothesis 2—more time spent on dating apps would be associated with poorer body image dimensions—we ran the same models as above, replacing time spent on social media with time spent on dating apps (*N* = 1254). Analyses were conducted using R. We used the lmer() and lmerTest() packages to test mixed‐effects regression models for our main analysis which used listwise deletion.

## RESULTS

### Preliminary analysis

Ninety‐four per cent of young adults (*N* = 5734) in this study used social media. The mean time spent on social media across the sample was a mean rating of 3.35 (SD = 1.34) on the 6‐point Likert scale, corresponding to approximately 6–7 h per week. Women spent more time on social media (M = 3.44, SD = 1.33) than men (M = 3.17, SD = 1.33), *t* = −6.89, df = 3353.10, *p* < .001. See Table 1 for a breakdown by country. In most countries, over 95% of individuals reported using social media (except for China), while use of dating apps varied between 5% and 45% of respondents.

The time spent on dating apps across the sample was a mean rating of 1.71 (SD = .92) on the 6‐point Likert scale, corresponding to approximately 1–2 h per week (see Table 1). Men spent more time on dating apps (M = 1.79, SD = .94) than women (M = 1.62, SD = .90), *t* = 3.05, df = 1052, *p* = .002.

A table outlining means, standard deviations and correlations among the demographics and the study variables is provided in the Supporting Information.

### Main analysis

#### Time spent on social media and body image

**FIGURE 1:**
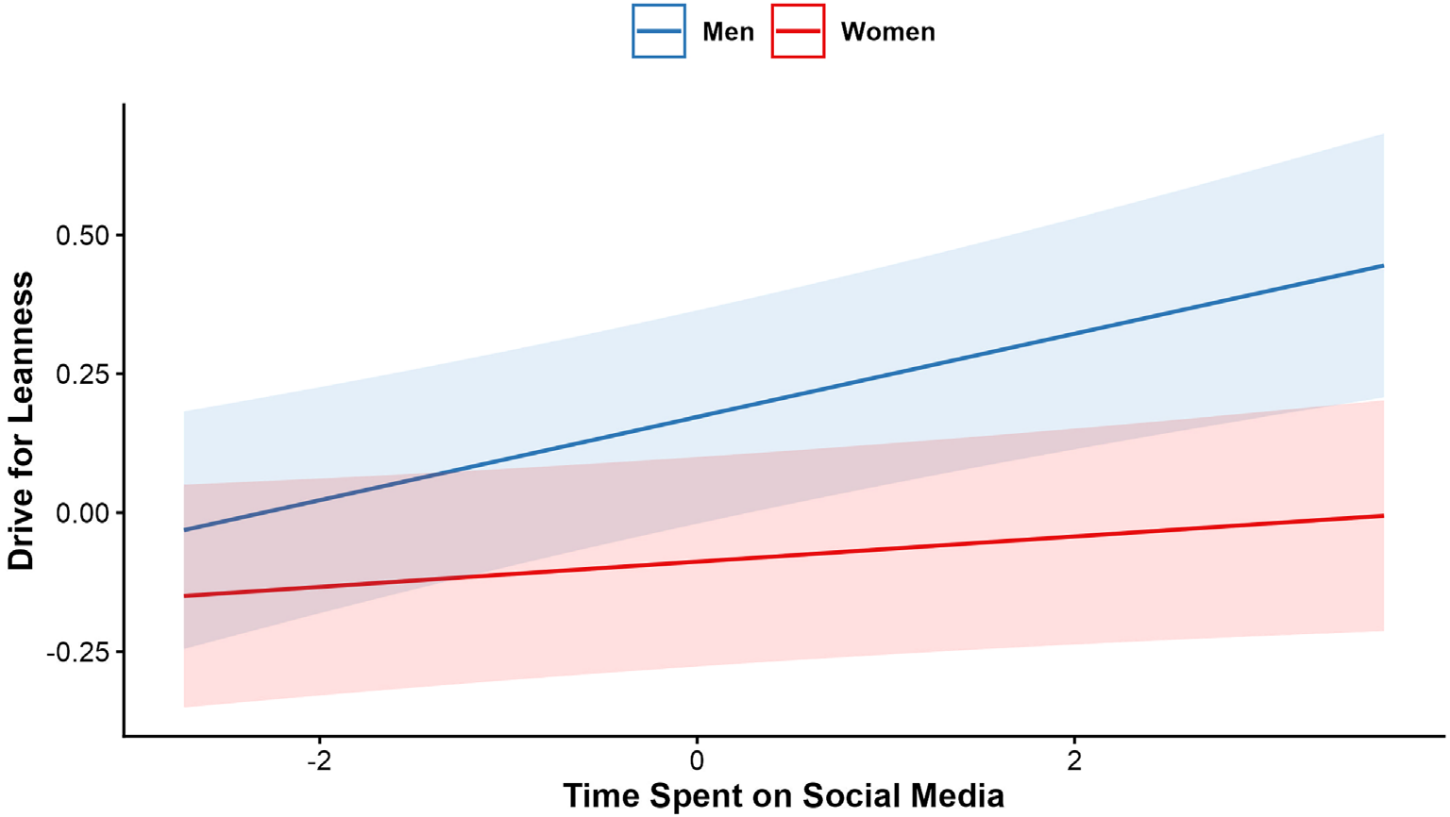
Association between time spent on social media and drive for leanness by gender. *Note*: Standardized effects.

**FIGURE 2:**
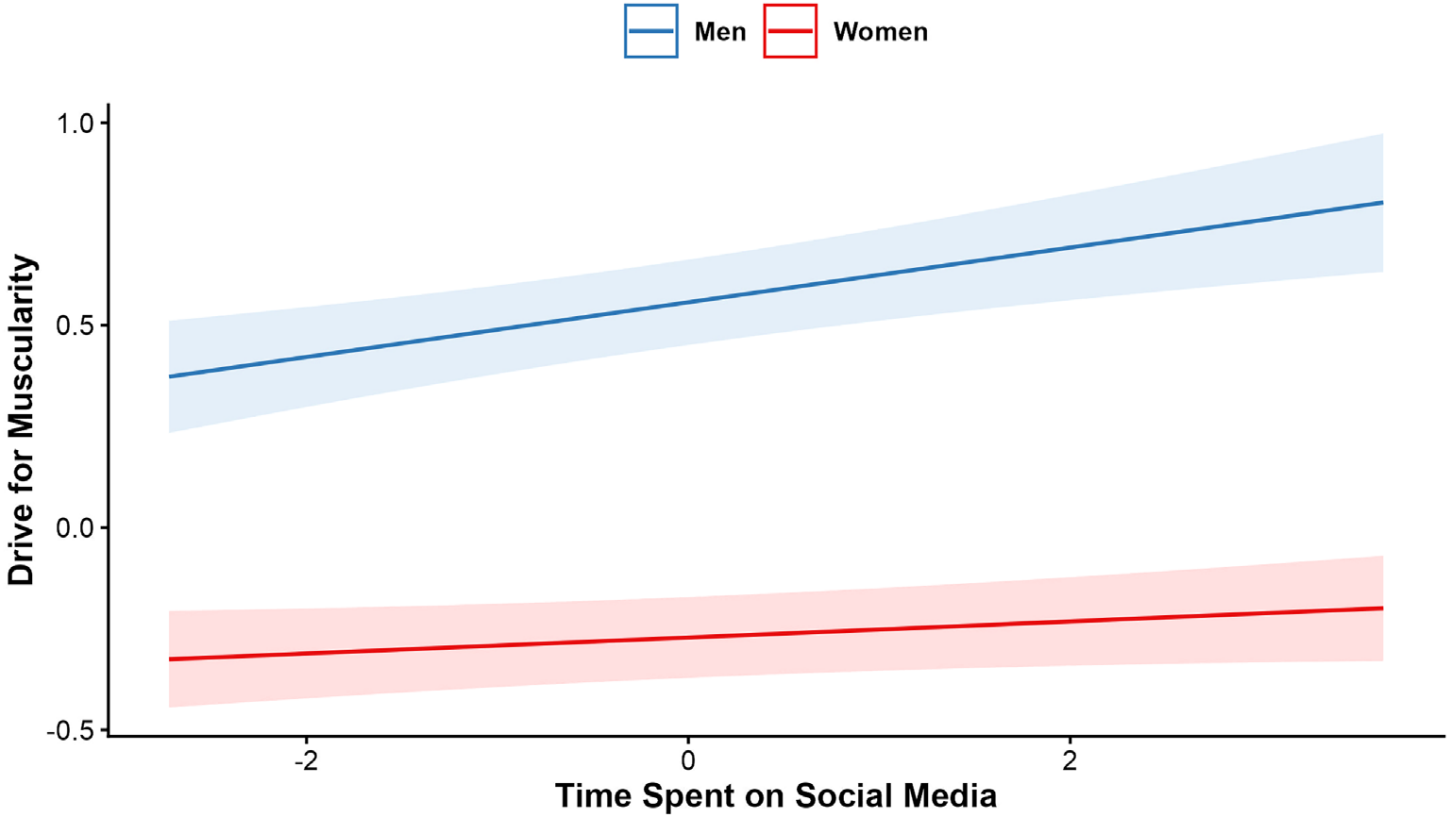
Association between time spent on social media and drive for muscularity by gender. *Note*: Standardized effects.

Regarding Hypothesis 1, more time spent on social media was significantly associated with lower scores on body satisfaction and body appreciation, *p* < .001, and higher scores on drive for muscularity, *p* < .001, drive for leanness, *p* < .004, internalization of the thin ideal and appearance comparison, *p*s < .001; however, we note that these effects were small, ranging from betas of .03 (drive for leanness) to .08 (appearance comparisons). The associations between time spent on social media and drive for muscularity and drive for leanness were stronger for men compared to women, *p* < .026 (see Table 2 and Figures 1 and 2), all other interactions were non‐significant. No significant interactions were found between time spent on social media and country. See Table 2 for detailed results.

**TABLE 2:** Results summary from mixed‐effects regression models testing the associations between time on social media and time on dating apps with measures of body satisfaction, including interactions with gender and country as omnibus effects.

Outcome	Predictor	β (95% CI)	SE	*p*	Interaction	Mean sq	df	*F*	*p*
Body satisfaction	Social media	−.06	.01	<.001	Social media × Gender	.84	15,392	1.03	.310
		(−.07, −.04)			Social media × Country	1.12	75,456	1.33	.231
	Dating apps	−.05	.03	.146	Dating apps × Gender	.12	11,027	.13	.721
		(−.12, .02)			Dating apps × Country	1.39	71,050	1.5	.162
Body appreciation	Social media	−.05	.01	<.001	Social media × Gender	.25	15,311	.31	.579
		(−.06, −.03)			Social media × Country	1.4	75,372	1.71	.102
	Dating apps	−.1	.03	.003	Dating apps × Gender	.19	11,008	.22	.642
		(−.17, −.04)			Dating apps × Country	.62	71,030	.71	.665
Drive for muscularity	Social media	.07	.02	<.001	Social media × Gender	4.11	15,108	4.95	.026
		(.03, .1)			Social media × Country	1.8	75,163	1.86	.071
	Dating apps	.14	.05	.008	Dating apps × Gender	5.2	1968	5.8	.016
		(.04, .24)			Dating apps × Country	2.44	7985	2.27	.027
Drive for leanness	Social media	.03	.01	.004	Social media × Gender	4.86	15,089	5.38	.020
		(.01, .05)			Social media × Country	1.56	75,147	1.69	.105
	Dating apps	0	.04	.993	Dating apps × Gender	1.71	1964	1.78	.183
		(−.07, .07)			Dating apps × Country	1.65	7983	1.68	.111
Thin idealization	Social media	.04	.01	<.001	Social media × Gender	.22	15,277	.26	.612
		(.02, .06)			Social media × Country	1.03	75,337	1.12	.349
	Dating apps	.02	.03	.557	Dating apps × Gender	1.67	11,006	1.91	.167
		(−.05, .09)			Dating apps × Country	.82	71,026	.88	.518
Appearance comparisons	Social media	.08	.01	<.001	Social media × Gender	.17	15,129	.2	.655
		(.06, .1)			Social media × Country	1.7	75,187	1.93	.061
	Dating apps	−.07	.03	.047	Dating apps × Gender	.69	1969	.88	.349
		(−.13, 0)			Dating apps × Country	1.23	7990	1.51	.161

*Note*: 95% CI, 95% confidence intervals for beta (lower, upper); Dating apps, time spent on dating apps; df, degrees of freedom; Mean Sq, mean square difference; SE, standard error; Social media, time spent on social media. Analyses controlled for BMI and age.

#### Time spent on dating apps and body image

**FIGURE 3:**
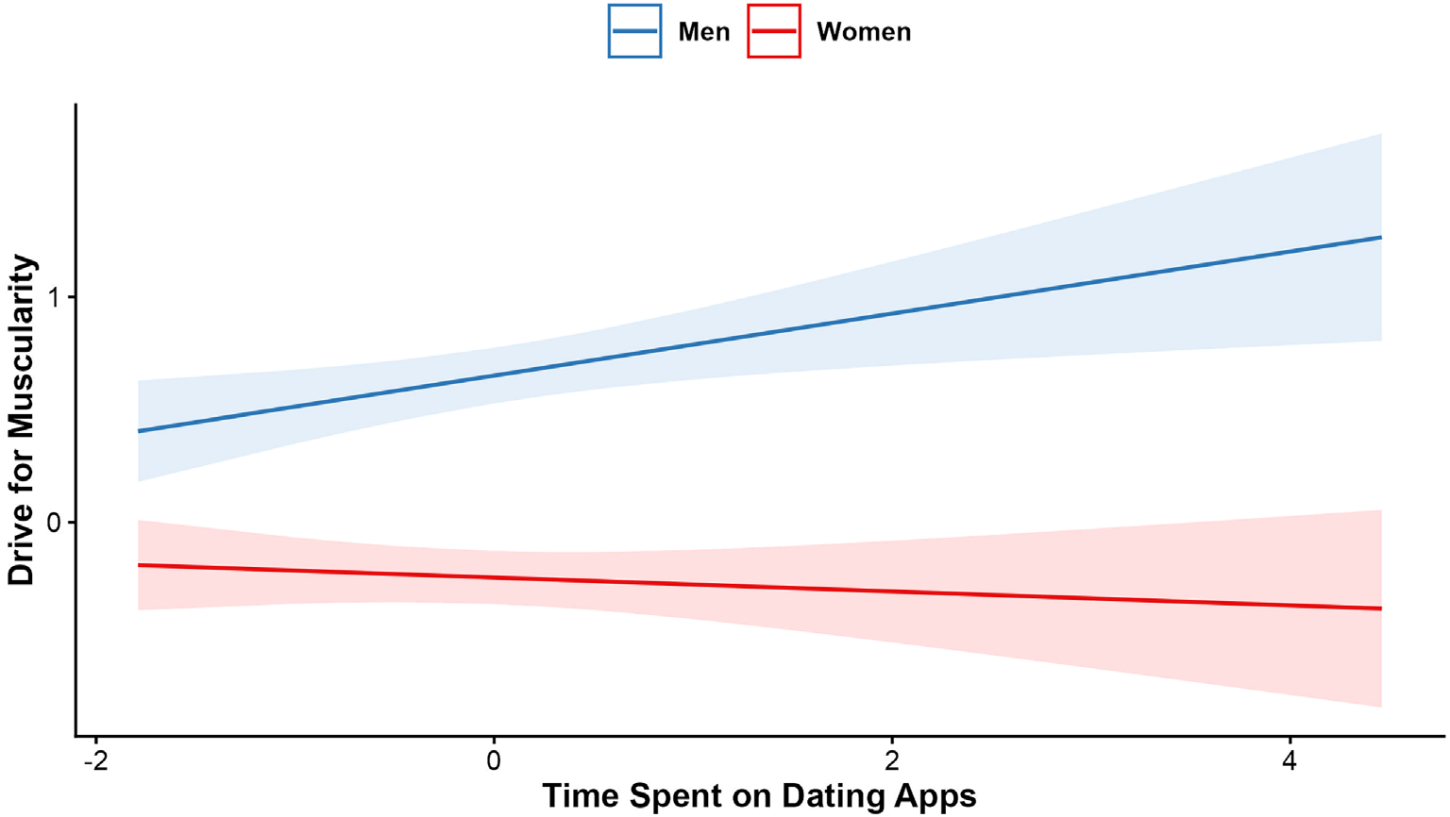
Association between time spent on dating apps and drive for muscularity by gender. *Note*: Standardized effects.

**FIGURE 4:**
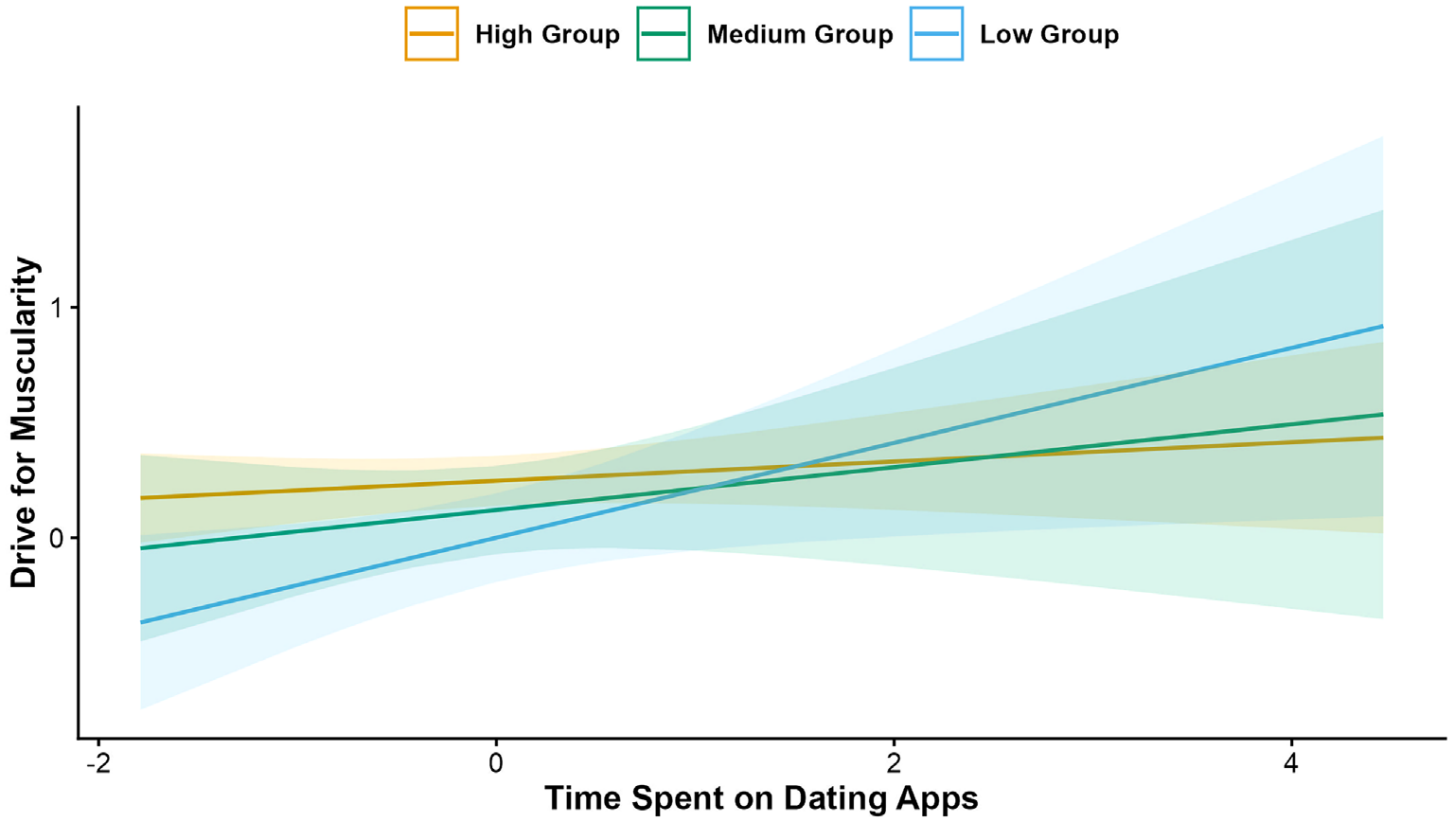
Association between time spent on dating apps and drive for muscularity by country group. *Note*: Standardized effects. Countries were grouped based on mean scores on Drive for Muscularity (DM). Countries in the High DM Group = Australia, China and the USA; Medium DM Group = Canada and Spain; and Low DM Group = Belgium, Italy and Japan.

Hypothesis 2 predicted that time spent on dating apps would predict body image constructs and, in line with this, the results showed that more time spent on dating apps was significantly associated with lower scores on body appreciation, *p* = .003, appearance comparison, *p* = .047 and higher scores on drive for muscularity, *p* = .008, again with small effect sizes, ranging from −.07 (appearance comparisons) to .14 (drive for muscularity). There were no significant associations between time spent on dating apps and body satisfaction, drive for leanness or internalization of the thin ideal, *p*s > .146. Regarding interaction terms, the associations between time spent on dating apps and drive for muscularity were stronger for men than women, *p* = .016 (see Figure 3). No significant interactions between time spent on dating apps and country were found, except for drive for muscularity, *p* = .027, see Table 2. Countries were first grouped based on scores on Drive for Muscularity (for country means). Countries in the ‘High’ group included China, Australia and the USA, countries in the ‘Medium’ group included Spain and Canada, and countries in the ‘Low’ group included Japan, Italy and Belgium. The association between time spent on dating apps and drive for muscularity was strongest among the Low group and weakest among the High group; however, pairwise comparisons revealed no significant differences between the groups (*p*s > .152) (see Figure 4).

## DISCUSSION

The aim of this study was to explore the associations between social media use, dating app use and different facets of poor body image. Overall, our findings supported our hypothesized associations between greater time spent on social media and poorer body image; however, these findings were not consistent across facets of body image and gender, with some relationships emerging more strongly among young men. The hypothesized relationships among dating app use and body image were only partially supported, however. In contrast, few differences were found across the countries examined. Together, these findings provide additional support for the presence of a cross‐sectional relationship among social media use, dating app use and poorer body image among young women and men, and the globalized nature of this relationship (Blake et al., 2022; de Valle et al., 2021; Strubel & Petrie, 2017).

The first hypothesis posited that greater social media use would be associated with poorer body image as assessed across different dimensions. Our findings supported this as higher time spent on social media was associated with lower body satisfaction and appreciation, and higher levels of appearance comparisons, drive for muscularity, drive for leanness and thin‐ideal internalization in the mixed gender sample. Moreover, interactions revealed that the relation between social media use and drive for muscularity was stronger among men than women. Similarly, the relationship between higher social media use and drive for leanness was stronger among men than women. In our sample, women reported spending more time on social media as compared to men overall; thus, it may be that either men who spend more time on social media are those who experience high levels of interest in, or concerns around, muscularity and leanness, or it may be that women's constant exposure to social media means that the relationships between social media use and body image outcomes are not tied to overall time spent on social media but more to the investment in photo‐based activities on those platforms (Bonfanti et al., 2025; de Valle et al., 2021; Lo Coco & Rodgers, 2025; McLean et al., 2015). Previous research suggested that women usually report lower scores in drive for leanness (and drive for muscularity) than men (e.g., Lang & Rancourt, 2020) and it is possible that different socially accepted stereotypes to which both groups tend to be more oriented (e.g., drive for muscularity vs drive for thinness) may play a role in social media use. The scoping review by Sharma and Vidal (2023) also outlined how males actively interact with social media content around fitspiration (e.g., content related to athletes and bodybuilders) as motivation to gain muscle, and that this shifts their focus from ‘health’ to appearance issues.

Less support was found for the second hypothesis, namely that higher time spent on dating apps would be associated with body image dimensions. Indeed, this relationship was confirmed only among two of the six indices of body image examined. Specifically, more time on dating apps was associated with lower body appreciation and higher drive for muscularity. Moreover, people who use dating apps more often are less likely to compare themselves to others; however, this effect was small (*p* = .046) and due to the multiple analyses run, this result should be interpreted with caution. No relationship between higher time spent on dating apps, body satisfaction, drive for leanness or thin‐ideal internalization emerged. Some of these findings seem counterintuitive in contrast with that reported by Strubel and Petrie (2017), who showed that Tinder users reported significantly higher levels of internalization, appearance comparisons and body shame than non‐users. However, it is worth noting that most of the previous findings are based on a comparison between dating app users and non‐users rather than the reported number of hours spent on the app (Portingale et al., 2022; Tran et al., 2019). One factor that may affect this relationship and was not controlled for in our study is relationship status. Indeed, previous research has reported that individuals who are currently in a relationship are less likely to use dating apps (Blake et al., 2022). Moreover, romantic involvement has been shown to be associated with body image (Laus et al., 2018). Thus, accounting for relationship status in future work would be important. It is also notable though that heterosexual women and men in the current study look at the opposite sex on dating apps, so they are likely not comparing their bodies to the opposite sex. Based on the current findings, it could be speculated that a higher use of dating apps for viewing potential romantic partners of the opposite sex may involve less same‐gender body comparison. However, further longitudinal research is needed to disentangle the direction of these effects.

Similar to the interaction effect found for social media use, the relationship between dating apps use and drive for muscularity was stronger among men as compared to women. This may reflect the centrality of muscularity in male appearance ideals and their concern to appear more muscular in order to be attractive to women (Sharma & Vidal, 2023; Watson et al., 2019). These findings may also be reflective of the ways in which dating app use may vary across intersections of identities, including among sexual minority men, who are also documented to have high levels of endorsement of muscularity ideals (Nowicki et al., 2022). Indeed, research has shown that sexual minority men who use dating apps experience strong appearance pressures on these platforms associated with body image concerns (Tran et al., 2020, 2023). While our study did not control for sexual orientation, disentangling these intersectional effects would be an important future direction.

We also investigated potential cross‐country variations in the magnitude of the relationships between social media and dating apps use and the different facets of body image. However, no significant differences were found across samples coming from different countries. This is an important finding, as the current study is one of the first to have examined these relationships in non‐English speaking countries using scales that had previously been validated in these contexts (Aimé et al., 2020; Rodgers, Fuller‐Tyszkiewicz, et al., 2020; Sicilia et al., 2020). In light of the pervasive use of social media on a global scale, the present findings contribute to the understanding of the relationship between social media use and body image across diverse national contexts. Given the large sample size this may reflect the effects of globalization, and absence of culturally specific protective factors in the relationship between social media use and body image. However, the lack of documented moderation effects may be related to the overall similar economic levels of the countries included, or the somewhat global assessment of social media use. It is indeed important to note that the countries included here were relatively affluent in English. It would be important to further diversify geographical areas to increase the understanding of the generalizability of these findings across the globe.

Moreover, future research should include additional characteristics on the country level that may be able to account for country differences. For example, our results suggest that participants from Spain and Italy reported less use of dating apps, and it could be speculated that a mild climate in combination with cultural norms around frequent socialization in public spaces allows for more outdoor socializing and that dating apps are not as widespread a substitute as they might be in other countries. Overall, our results showed that the use of dating apps largely varied across countries, and it is likely that a variety of cultural values can influence individual relationship behaviours and their relation to body image, such as the dimensions of masculinity–femininity, power‐distance and individualism (Hofstede, 2001).

These findings have several implications. First, they provide further support that social media use is associated with poor body image (Saiphoo & Vahedi, 2019; Vlasak et al., 2025) and extends this finding across genders, even when using a somewhat rudimentary measure of time spent on social media (cf. Jarman et al., 2022). The robustness of these findings across contexts and genders speaks to the importance of this issue, even though our study utilized a cross‐sectional design. Furthermore, the suggestion that these relationships may be even stronger for some body image dimensions among young men as compared to young women is particularly important in light of the relative neglect of young men in this area of research. However, the over‐reliance on female participants in the present study confirms that the involvement of participants who identify as men is challenging and that further work is needed to address the specific barriers that are likely due to the social roles and norms. Additional work focused on understanding the ways in which social media and dating apps use can increase risk for body image concerns, especially among young men and not just young women is important. Finally, the implications of these relationships replicating across cultural contexts highlight the potential negative effects of social media use among youth on a global scale, and their potential for increasing appearance concerns worldwide (Rodgers et al., 2023). This expansion calls for widespread attention to social media literacy and education and programming surrounding the use of social media, as well as increased attention to policies and practices that can help to decrease the presence of potential harmful content or negative experiences on social media.

This study includes several noteworthy limitations. First, while the measures of body image were well established, the measures of social media and dating apps use were not fine‐grained. Additional work using measures capable of capturing the different patterns of use across users, as well as the different types of content that people engage with, would offer greater insights into these relationships (Valkenburg, 2022). Recent evidence has indicated that the duration of time spent on social media, or the breadth of accessed platforms, is not associated with body image variables. Instead, the type of content engaged with (e.g., weight loss content exposure) has been identified as a significant factor (Sanzari et al., 2023). With regards to dating apps, previous evidence found a dearth of research on how specific features or characteristics of dating apps can affect users' body image differently (Bowman et al., 2025). Additionally, there is growing evidence of a weak relationship between single estimate self‐report social media use and objective measure of social media use (Burnell et al., 2021; Mahalingham et al., 2023). Future research should seek to reduce the reliance on self‐report measures that have dominated the field, towards the adoption of more objective and content‐based measures of social media or dating app use. In addition, social media and dating platforms evolve quickly, which may limit the generalizability of findings as the platforms change more quickly than the research can keep up. In our analyses, gender was considered dichotomously, and it would be worthwhile to consider in future studies a broader categorization of gender and sexual orientation, as well as considering relationship status. Dating apps use seems particularly high for people who identify as lesbian, gay or bisexual (Pew Research Center, 2023), but the research among this population is still limited. Furthermore, the majority of participants in our sample identified as female, although there were variations in the regional distribution of the data. It is imperative that future cross‐gender surveys on social media and body image develop innovative strategies to gather more balanced samples in terms of gender identities. It is worth noting that both time of use and mode of use of social media and dating apps may vary with age and be different at different life stages. Future studies should extend the examination of these relationships at different life stages. Finally, the data were cross‐sectional which precludes us from making any inferences as to causality. Longitudinal work, as well as experimental work focused on dating apps in particular, would help to better characterize the directionality of the relationships between digital platform use and body image.

In sum, the present study supports and extends previous work documenting a relationship between greater time spent on social media and dating apps and poorer body image to both young women and men, across eight different countries. Additional studies are warranted to better understand these relationships, identify those most at risk of potential negative effects of the use of these platforms and inform prevention and intervention efforts that may mitigate the possibly negative consequences of social media and dating app use.

## AUTHOR CONTRIBUTIONS

**Gianluca Lo Coco:** Conceptualization; data curation; writing – original draft; methodology. **Rachel Rodgers:** Conceptualization; investigation; methodology; writing – original draft. **Emily A. Harris:** Methodology; formal analysis. **Charlotte Markey:** Investigation; supervision; writing – review and editing. **Alvaro Sicilia:** Methodology; writing – review and editing. **Annie Aimé:** Data curation; supervision; writing – review and editing. **Jacinthe Dion:** Writing – review and editing. **Laura Salerno:** Formal analysis; data curation. **Naomi Hayami‐Chisuwa:** Data curation; writing – review and editing. **Hannah J. White:** Writing – review and editing. **Carolyn R. Plateau:** Writing – review and editing. **Antonio Granero‐Gallegos:** Writing – review and editing. **Christophe Maïano:** Methodology; writing – review and editing. **Gian Mauro Manzoni:** Writing – review and editing. **Giada Pietrabissa:** Writing – review and editing. **Catherine Bégin:** Writing – review and editing. **Marie‐Éve Blackburn:** Writing – review and editing. **Esben Strodl:** Writing – review and editing. **Matthew Fuller‐Tyszkiewicz:** Supervision; methodology; formal analysis. **Marita McCabe:** Supervision; writing – review and editing.

## CONFLICT OF INTEREST STATEMENT

The authors report no conflict of interest.

## Supporting information


Data S1

## Data Availability

The data that support the findings of this study are available on request from the corresponding author. The data are not publicly available due to privacy or ethical restrictions.
