# Supplementary material for: Investigating the relation between social media, dating app use and body image dimensions: A cross‐country study
Source: Br J Health Psychol. 2026 Mar 30;31(2):e70067. doi: 10.1111/bjhp.70067 (PMC13034889; doi:10.1111/bjhp.70067)
Supplement: Supplementary file 1 — Data S1 [file BJHP-31-0-s001.docx]

Means, Standard Deviations, and Correlations

| Variable | Mean | SD | 1 | 2 | 3 | 4 | 5 | 6 | 7 | 8 | 9 | 10 |
| --- | --- | --- | --- | --- | --- | --- | --- | --- | --- | --- | --- | --- |
| 1. Age | 21.54 | 3.13 | — |  |  |  |  |  |  |  |  |  |
| 2. BMI | 23.30 | 4.94 | 0.16*** | — |  |  |  |  |  |  |  |  |
| 3. Social MediaTime | 3.35 | 1.34 | -0.07*** | 0.02 | — |  |  |  |  |  |  |  |
| 4. Dating Time | 1.71 | 0.92 | 0.01 | 0.04 | 0.09** | — |  |  |  |  |  |  |
| 5. Body Satisfaction | 3.19 | 0.74 | 0.03* | -0.17*** | -0.04** | -0.06 | — |  |  |  |  |  |
| 6. Body Appreciation | 3.46 | 0.94 | 0.02 | -0.13*** | -0.04*** | -0.07* | 0.75*** | — |  |  |  |  |
| 7. Drive Muscle | 2.18 | 0.92 | 0.03* | 0.01 | -0.01 | 0.05 | -0.07*** | -0.05*** | — |  |  |  |
| 8. Drive Lean | 21.75 | 7.21 | 0.06*** | 0.04** | 0.03* | 0.01 | -0.08*** | -0.04** | 0.40*** | — |  |  |
| 9. Intern Thin | 3.05 | 1.00 | -0.07*** | 0.08*** | 0.04** | 0.03 | -0.38*** | -0.37*** | 0.02 | 0.30*** | — |  |
| 10. Appearance Comparisons | 14.98 | 4.24 | 0.04** | 0.07*** | 0.09*** | -0.11*** | -0.40*** | -0.39*** | 0.09*** | 0.32*** | 0.44*** | — |

Note: Variables were uncentered. Correlations use pairwise deletion. * p < .05, ** p < .01, *** p < .001.
